# Cryptorchidism and testicular germ cell tumors: comprehensive meta-analysis reveals that association between these conditions diminished over time and is modified by clinical characteristics

**DOI:** 10.3389/fendo.2012.00182

**Published:** 2013-02-18

**Authors:** Kimberly Banks, Ellenie Tuazon, Kiros Berhane, Chester J. Koh, Roger E. De Filippo, Andy Chang, Steve S. Kim, Siamak Daneshmand, Carol Davis-Dao, Juan P. Lewinger, Leslie Bernstein, Victoria K. Cortessis

**Affiliations:** ^1^Keck School of Medicine, University of Southern CaliforniaLos Angeles, CA, USA; ^2^City of Hope National Medical CenterDuarte, CA, USA; ^3^St. Joseph HospitalOrange, CA, USA; ^4^Children's Hospital Los AngelesLos Angeles, CA, USA

**Keywords:** testicular neoplasms, cryptorchidism, seminoma, non-seminoma, meta-analysis

## Abstract

**Introduction:** Risk of testicular germ cell tumors (TGCT) is consistently associated with a history of cryptorchidism (CO) in epidemiologic studies. Factors modifying the association may provide insights regarding etiology of TGCT and suggest a basis for individualized care of CO. To identify modifiers of the CO-TGCT association, we conducted a comprehensive, quantitative evaluation of epidemiologic data.

**Materials and Methods:** Human studies cited in PubMed or ISI Web of Science indices through December 2011 and selected unpublished epidemiologic data were reviewed to identify 35 articles and one unpublished dataset with high-quality data on the CO-TGCT association. Association data were extracted as point and 95% confidence interval estimates of odds ratio (OR) or standardized incidence ratio (SIR), or as tabulated data. Values were recorded for each study population, and for subgroups defined by features of study design, CO and TGCT. Extracted data were used to estimate summary risk ratios (sRR) and evaluate heterogeneity of the CO-TGCT association between subgroups.

**Results:** The overall meta-analysis showed that history of CO is associated with four-fold increased TGCT risk [RR = 4.1(95% *CI* = 3.6–4.7)]. Subgroup analyses identified five determinants of stronger association: bilateral CO, unilateral CO ipsilateral to TGCT, delayed CO treatment, TGCT diagnosed before 1970, and seminoma histology.

**Conclusions:** Modifying factors may provide insight into TGCT etiology and suggest improved approaches to managing CO. Based on available data, CO patients and their parents or caregivers should be made aware of elevated TGCT risk following orchidopexy, regardless of age at repair, unilateral vs. bilateral non-descent, or position of undescended testes.

## Introduction

Cryptorchidism (CO), or undescended testis, affects approximately 3% of all male live births, making it one of the most common congenital disorders. Despite a high rate of spontaneous resolution during the first year of life, CO is firmly established as the primary risk factor for subsequent development of testicular germ cell tumors (TCGT) (John Radcliffe Hospital Study Group, 1992; Berkowitz et al., 1993; Thong et al., 1998; Paulozzi, 1999). TGCT are the most common form of malignancy among young men in the United States. Advances in systemic therapy have improved overall TGCT survival from 83% in 1975–1979 to 96% in 1999–2005 (Jemal et al., 2010). However, incidence of TGCT has nearly doubled during the same time period (Fast Stats), and it is now evident that significant sequelae include subfertility, (Walsh et al., 2009) sexual dysfunction, (Magelssen et al., 2006) and elevated risk of second malignancy (Moller et al., 1993; Fossa et al., 2005; Travis et al., 2005; Van den Belt-Dusebout et al., 2007). Therefore, a clear understanding of etiologic risk factors and more comprehensive risk stratification is a priority of TGCT research.

Risk of CO and TGCT are associated with additional disorders of the male reproductive system, hypospadias, and impaired spermatogenesis. This pattern is postulated to reflect origins of all of these conditions in errors of development of the fetal testis according to the testicular dysgenesis syndrome hypothesis, which elegantly accounts for experimental research identifying genetic and early environmental factors predisposing to these phenotypes in animal models (Skakkebæk et al., 2001). Little is currently known regarding the specific insults that may lead to elevated risk of individual and joint phenotypes in humans, or the stages of testicular development when such factors may act. In the present report we comprehensively reviewed the rich set of published observational data on co-occurrence of CO and TGCT as a first step in disentangling the complex associations among these related conditions.

Currently, little is known about the overall characterization of tumor risk in patients with a prior history of CO. Individual estimates of relative risk from the literature range from 1.35 (95% *CI* = 0.73–2.48) to 18 (95% *CI* = 12–26), (Miller and Seljelik, 1971; Mostofi, 1973; Morrison, 1976; Henderson et al., 1979; Loughlin et al., 1980; Schottenfeld et al., 1980; Wobbes et al., 1980; Fonger et al., 1981; Coldman et al., 1982; Depue et al., 1983; Mills et al., 1984; Pottern et al., 1985; Moss et al., 1986; Giwercman et al., 1987; Swerdlow et al., 1987, 1997; Gershman and Stolley, 1988; Strader et al., 1988; Thornhill et al., 1988; Haughey et al., 1989; Benson et al., 1991; Stone et al., 1991; United Kingdom Testicular Cancer Study Group (UK), 1994; Gallagher et al., 1995; Davies et al., 1996; Moller et al., 1996; Prener et al., 1996; Petridou et al., 1997; Sabroe and Olsen, 1998; Sigurdson et al., 1999; Weir et al., 2000; Stang et al., 2001; Bonner et al., 2002; Herrinton et al., 2003; Dieckmann and Pichlmeier, 2004; Kanto et al., 2004; Hardell et al., 2007; McGlynn et al., 2007; Myrup et al., 2007; Pettersson et al., 2007; Walschaerts et al., 2007; Dusek et al., 2008) suggesting significant differences in study design and/or heterogeneity of the effects of clinical characteristics which may impact risk of TGCT amongst males with CO. Potential modifying factors include anatomic location (abdominal vs. inguinal vs. ectopic) and laterality (unilateral vs. bilateral) of undescended testes, age at treatment, mode of treatment (spontaneous descent vs. hormones or orchiopexy), as well as temporal trends in TGCT risk, and tumor histology. Previous meta-analyses evaluating the CO-TGCT association have been limited to specific subsets of these factors (Castejon Casado et al., 2000; Walsh et al., 2007; Tuazon et al., 2008; Akre et al., 2009). We report a broader systematic review and meta-analysis of the overall association between CO-TGCT, and explore the possible impact of study design, temporal trends, and clinical features on this association.

## Materials and methods

The analysis followed specifications for meta-analysis of observational studies in epidemiology (Stroup et al., 2000) and adhere to PRISMA guidelines (Moher et al., 2009). The outcome was TGCT. The exposure was CO, defined as a testicle undescended at birth that subsequently descended spontaneously, was repositioned into the scrotum by orchiopexy or hormone therapy, or remained undescended. Subgroups were defined by features of study design, CO, and TGCT.

### Study selection

We searched the MEDLINE (National Library of Medicine, Bethesda, MD, USA) Pubmed interface without language restrictions for human studies published through December 2011, using key words “CO,” “undescended testicle,” “undescended testis,” “undescended testes,” “case-control study,” “cohort study” in combination with “testicular cancer,” “testicular carcinoma,” “testicular neoplasia,” “testis cancer,” “testis carcinoma,” “testis neoplasia,” reviewing also reports cited in retrieved articles and review articles, and by citation indices (ISI Web of Science) for these reports. We also sought high-quality unpublished data. The Review Protocol has not been registered.

### Data extraction and coding

A genetic counselor and a statistician reviewed retrieved articles to determine eligibility for the meta-analysis, resolving conflicts by consensus among themselves and an epidemiologist. Data on study design, overall CO-TGCT associations, and subgroup-specific CO-TGCT associations were extracted systematically by a single reviewer and confirmed by two others. We eliminated redundant data arising from repeated publication, consulting original authors whenever possible.

We extracted published information on RRs relating CO to TGCT as follows: when provided, we recorded point estimates of the odds ratio (OR) for case-control studies and standardized incidence ratio (SIR) for cohort studies, with corresponding standard error or information from which it could be calculated (variance, *CI*, *p*-value). When only a point estimate was reported, we requested corresponding variance term from original authors. If the OR estimate was not provided, we calculated it from published tabular data.

#### Features of study design

We noted the following features of each study: data structure (case-control study of TGCT, cohort study of males born with CO, TGCT cases for whom frequency of CO was compared to external population), country where study was conducted, race/ethnicity of participants, and source of CO data (birth record, medical record before TGCT diagnosis, medical record at/following TGCT diagnosis, reported by participant and/or his mother). For case-control and cohort studies we noted source of reference group (population or population-based registry, hospital or neighborhood, friend). We distinguished published RR estimates extracted from those we calculated from published data; for published estimates, we tabulated covariates and matching variables in original analyses.

#### Features of cryptorchidism

When possible we extracted or calculated RR estimates of CO-TGCT association for subgroups defined by each of several features of CO: laterality of undescended testicle relative to TGCT (unilateral CO contralateral to tumor, unilateral CO with relation to tumor unspecified, unilateral CO ipsilateral to tumor, bilateral CO), level of maldescent (ectopic, inguinal, abdominal), whether definition of CO included spontaneous descent, means of resolving CO (spontaneous, orchiopexy or hormone therapy, remained undescended), and age at resolution. For this variable we used frequently published categories (0–9 years of age, 10–14 years, 10 years or older, 15 years or older) (United Kingdom Testicular Cancer Study Group (UK), 1994; Moller et al., 1996; Swerdlow et al., 1997) combining data from smaller intervals if provided (Swerdlow et al., 1997). Among studies with alternate cut points, one provided raw data from which we recalculated estimates within above strata (Herrinton et al., 2003); for four others (Pottern et al., 1985; Strader et al., 1988; Myrup et al., 2007; Pettersson et al., 2007) we assigned data to closest category corresponding to above strata (**Table S3**, footnotes b–d).

#### Features of testicular germ cell tumors

We extracted or calculated RR estimates for subgroups defined by histology (non-seminoma, non-seminoma/mixed germ cell tumor (GCT), mixed GCT, seminoma). A separate code for mixed GCT was introduced in 1990 (ICD-0-2), and tumors of mixed histology were previously coded as non-seminoma. Therefore, for studies including diagnoses before 1990, if authors did not specify that non-seminoma excluded mixed histology, we coded reported non-seminoma as “non-seminoma/mixed GCT.” As a measure of year of TGCT diagnosis, we determined midpoint of range of years of diagnosis among cases participating in each study.

#### Unpublished data

Original population-based case-control data (Lacson et al., 2012) were provided before publication. Briefly, 163 TGCT cases identified by the Los Angeles County Cancer Surveillance Program (CSP) and 284 age-matched neighborhood controls were enrolled. TGCT data (histologic type, laterality, age at diagnosis) were provided by the CSP, and CO data (laterality, age at resolution, mean of resolution) were provided by participants and their mothers during in-person interviews.

### Statistical analyses

#### Estimating overall and stratum-specific summary RR of CO-TGCT association

We conducted all meta-analyses using a random effects model, which accounts for between- and within-study variance thereby incorporating the conservative assumption that individual studies estimate different effect sizes (Sutton et al., 2000). We used STATA 8.0 (Stata Corp, College Station, TX) to weight natural log of each contributing OR or SIR estimate by the reciprocal of the corresponding variance. We used this technique to estimate overall and stratum-specific summary relative risk (sRR) estimates. Forest plots were graphed displaying each study's contribution to sRRs.

**Figure 1:**
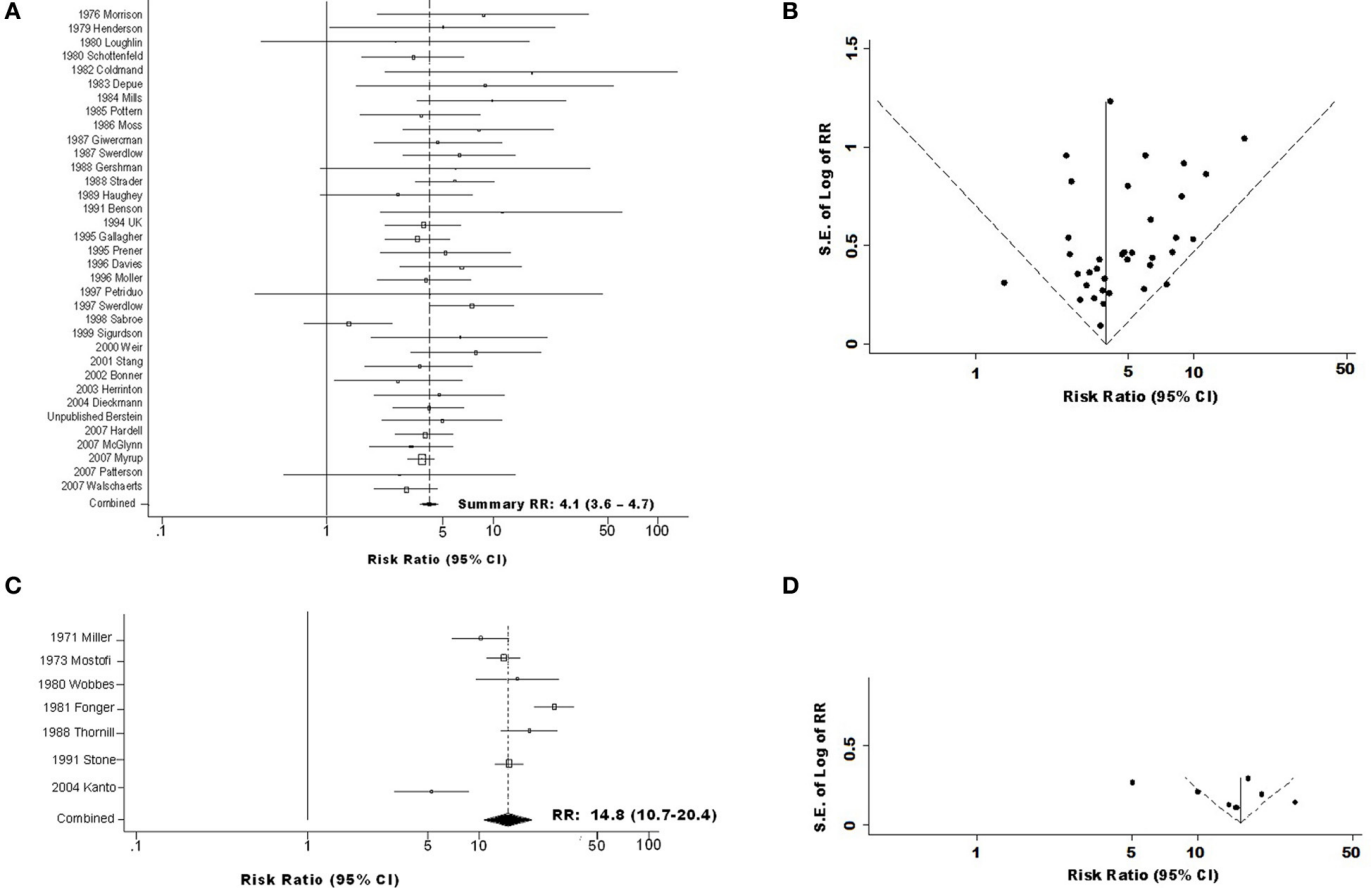
**Forest and funnel plots for overall association of testicular germ cell tumor (TGCT) risk with history of cryptorchidism (A) Forest plot for combined cohort and case-control data; (B) Funnel plot for combined cohort and case-control data; (C) Forest plot for TGCT case series data; (D) Funnel plot for TGCT case series data**.

**Figure 2:**
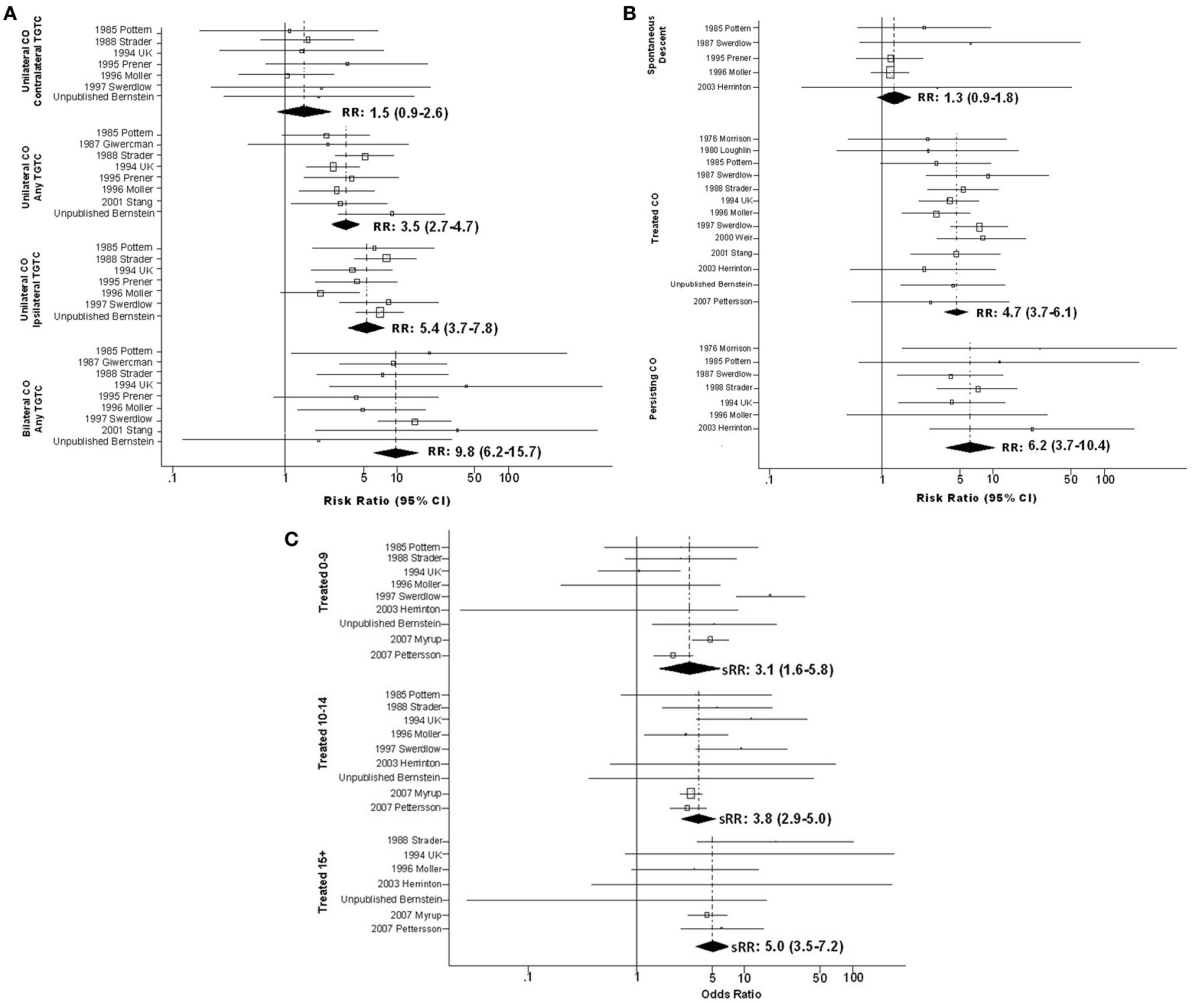
**Forest plots of relative risk estimates relating cryptorchidism to risk of TGCT, stratified by features of cryptorchidism; (A) By laterality of cryptorchidism relative to tumor; (B) By method whereby cryptorchidism was resolved; (C) By age at treatment for cryptorchidism**.

**Figure 3:**
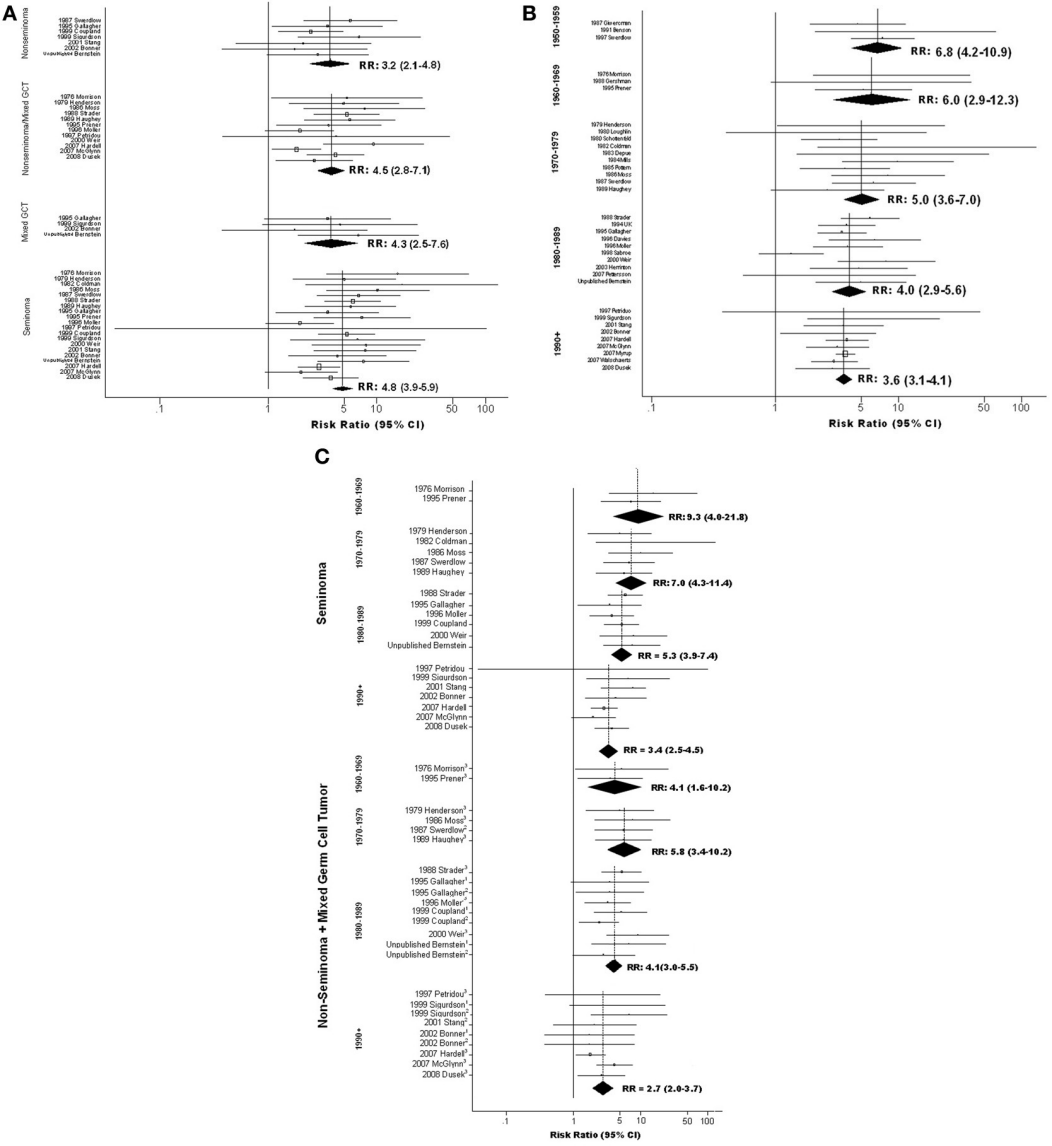
**Forest plots of relative risk estimates relating cryptorchidism to risk of TGCT, stratified by features of TGCT; (A) By histologic type of tumor; (B) By year of TGCT diagnosis (midpoint for study); (C) By histologic type of tumor and year of TGCT diagnosis (midpoint for study)**. ^1^Mixed; ^2^Non-Seminoma; ^3^Non-seminoma+Mixed.

Meta-analyses were performed separately for case-control (**Tables S2–S4**, columns A) and cohort (**Tables S2–S4**, columns B) studies, with summary measures subsequently pooled. Resulting sRR estimates [Figures 1A, 2A–C and 3A–C; **Tables S2–S4**, columns C (boldface)] summarize available data on CO-TGCT associations from studies with reference groups judged comparable to cases. Other articles compared CO prevalence of TGCT cases with CO prevalence from external populations, rather than source population of the cases; limited comparability in such studies is now recognized as a potential source of severe bias (Rothman et al., 2008). We therefore summarized these data separately (Figure 1C; **Tables S2–S4**, column D).

We implemented Egger's test of publication bias, assessed heterogeneity by appropriate *p*-value and I^2^ (Berkowitz et al., 1993) estimates, and assessed trends as described in Appendix Methods, along with procedures for imputation and sensitivity analysis.

### Role of the funding source

Sponsors had no role in the design, implementation, or reporting of the research. Drs. Victoria K. Cortessis and Leslie Bernstein had full access to all of the original data in the study and take responsibility for the integrity of the data and the accuracy of the data analysis.

## Results

We identified 147 published articles reporting on both CO and TGCT in humans, eliminating those that did not provide data relating CO to TGCT risk (*N* = 95, not cited), or were duplicate reports of data included in the analysis (10 reports, **Table S1**). Data from 35 of the remaining 42 reports and raw data from one unpublished case-control study contributed to meta-analyses on which we base our inferences (Table 1A; **Figure S1**). We requested from authors of three of these reports (Moss et al., 1986; Strader et al., 1988; Swerdlow et al., 1987) standard error data corresponding to published histology stratum-specific point estimates, and received these for one (Strader et al., 1988). In all, data on 9542 TGCT cases contributed to the analyses. For historical interest, we separately summarized estimates from the remaining seven reports, whose authors compared CO among TGCT case-series with frequencies of CO measured outside source populations of the cases (Table 1B).

**Table 1:** **Epidemiologic studies of cryptorchidism and testicular germ cell tumors (TGCT) included in the meta-analysis**.

**First author**	**Year published**	**Country**	**Ethnicity^a^**	**Number of TGCT cases**	**Number of controls**	**Source of cases^b^**	**Source of controls^b^**	**Spontaneous descent included^c^**	**Number of variables matched and/or adjusted for**	**Source of cryptorchidism data^d^**
**A. STUDIES THAT PROVIDED DATA ON TGCT CASES AND COMPARABLE REFERENCE GROUP**										
***i. Case-control studies***										
Morrison (Morrison, 1976)	1976	USA	NS	596	602	Reg	Pop	No	0	TCMR
Henderson (Henderson et al., 1979)	1979	USA	WP	79	79	Reg	Neigh	NS	2	M
Loughlin (Loughlin et al., 1980)	1980	USA	WO	24	35	Hosp	Hosp	No	2	C + M
Schottenfold (Schottenfeld et al., 1980)	1980	USA	WO	157	309	Hosp	Hosp + Neigh	NS	3	C + M
Coldman (Coldman et al., 1982)	1982	Canada	NS	93	79	Hosp	Hosp	Yes	2	C, TCMR
Depue (Depue et al., 1983)	1983	USA	WO	107	108	Reg	Neigh	NS	2	C
Mills (Mills et al., 1984)	1984	USA	WP	347	347	Hosp	Hosp	NS	3	TCMR
Pottern (Pottern et al., 1985)	1985	USA	WP	271	259	Hosp	Hosp	Yes	2	C + M
Moss (Moss et al., 1986)	1986	USA	WP	246	252	Reg + Hosp	Friends	NS	3	C, M
Swerdlow (Swerdlow et al., 1987)	1987	England	NS	259	489	Reg + Hosp	Hosp	Yes	2	C
Gershman (Gershman and Stolley, 1988)	1988	USA	NS	79	79	Reg	Pop	NS	2	C
Strader (Strader et al., 1988)	1988	USA	WO	326	675	Reg	Pop	No	1	C
Haughey (Haughey et al., 1989)	1989	USA	WO	247	247	Reg	Neigh	NS	2	C
UK (United Kingdom Testicular Cancer Study Group (UK), 1994)	1994	UK	WO	794	794	Reg + Hosp	Pop	No	1	C + M
Gallagher (Gallagher et al., 1995)	1995	Canada	WP	506	994	Reg	Pop	NS	1	C
Prener (Prener et al., 1996)	1996	Denmark	NS	171	366	Reg	Pop	Yes	4	PTCMR
Davies (Davies et al., 1996)	1996	England	NS	129	395	Reg	Pop + Reg	NS	6	C + M
Møller (Moller et al., 1996)	1996	Denmark	NS	514	720	Reg	Pop	Yes	1	C
Petridou (Petridou et al., 1997)	1997	Greece	NS	97	198	Hosp	Pop	NS	2	C + M
Sabroe (Sabroe and Olsen, 1998)	1998	Denmark	NS	357	704	Reg	Pop	Yes	2	BR
Sigurdson (Sigurdson et al., 1999)	1999	USA	WP	160	136	Hosp	Friends	NS	4	C
Weir (Weir et al., 2000)	2000	Canada	NS	325	490	Reg	Pop	No	5	C
Stang (Stang et al., 2001)	2001	Germany	NS	262	797	Reg	Pop	Yes	2	C, M
Bonner (Bonner et al., 2002)	2002	USA	WO	116	328	Hosp	Hosp	NS	1	C
Herrinton (Herrinton et al., 2003)	2003	USA	WP	183	551	Hosp	Hosp	Yes	2	PTCMR
Dieckmann (Dieckmann and Pichlmeier, 2004)	2004	Unknown	Unknown	538	551	Unknown	Unknown	Unknown	Unknown	Unknown
Hardell (Hardell et al., 2007)	2007	Sweden	NS	888	870	Reg	Pop	NS	1	C
McGlynn (McGlynn et al., 2007)	2007	USA	WP	767	928	Reg	Pop	NS	3	C
Walschaerts (Walschaerts et al., 2007)	2007	France	NS	200	585	Hosp	Hosp	NS	1	C
Dusek (Dusek et al., 2008)	2008	Czech Republic	WO	356	317	Hosp	Hosp + Neigh	NS	1	C
Lacson (Lacson et al., 2012)		USA	WP	163	284	Reg	Neigh	Yes	3	C
***ii. Cohort studies of boys with Cryptorchidism***										
Giwercman (Giwercman et al., 1987)	1987	Denmark	NS	6	–	Hosp	–	No	2	PTCMR
Benson (Benson et al., 1991)	1991	USA	NS	2	–	Hosp	–	Yes	1	PTCMR
Swerdlow (Swerdlow et al., 1997)	1997	England	NS	11	–	Hosp	–	No	2	PTCMR
Pettersson (Pettersson et al., 2007)	2007	Sweden	NS	56	–	Reg	–	No	2	PTCMR
Myrup (Myrup et al., 2007)	2007	Denmark	NS	110	–	Reg	–	No	2	PTCMR
**B. STUDIES THAT PROVIDED DATA ON TGCT CASE SERIES, ESTIMATING RISK RATIOS USING CRYPTORCHIDISM FREQUENCY FROM EXTERNAL POPULATION**										
Miller (Miller and Seljelik, 1971)	1971	Norway	NS	314	–	Reg	–	NS	–	TCMR
Mostofi (Mostofi, 1973)	1973	USA	NS	2000	–	Army Reg	–	NS	–	TCMR
Wobbes (Wobbes et al., 1980)	1980	Netherlands	NS	230	–	Hosp	–	No	–	TCMR
Fonger (Fonger et al., 1981)	1981	Canada	NS	646	–	Hosp	–	Yes	–	TCMR
Thornhill (Thornhill et al., 1988)	1988	Ireland	NS	240	–	Reg	–	NS	–	TCMR
Stone (Stone et al., 1991)	1991	Australia	NS	778	–	Hosp	–	Yes	–	TCMR
Kanto (Kanto et al., 2004)	2004	Japan	J	240	–	Hosp	–	Yes	–	TCMR

a *NS, not specified; WO, white only; WP, white plus other ethnicities; J, Japanese*.

b *Reg, cancer registry; Hosp, hospital; Neigh, neighborhood; Pop, population*.

c *NS, not specified*.

d *C, case/control self report; M, case/control mother's report; C + M, case/control self report supplemented by mother's report; BR, birth record; PTCMR, pre-testicular cancer medical record, TCMR: testicular cancer medical record*.

Sensitivity analyses revealed that no single case-control or cohort study influenced either overall or stratum-specific estimates of RR sufficiently to alter interpretation (results not shown). Egger's tests revealed no evidence of publication bias among cohort data (*p* = 0.68), case-control data (*p* = 0.34), or these data types combined (*p* = 0.40); however, addition of data from the TGCT case series introduced an impression of substantial publication bias (*p* = 0.01). Visual inspection of Begg's funnel plots showed that while magnitude and standard error of RR estimates from most cohort and case-control studies (34 of 36) are within the 95% confidence limits (Figure 1B), this is true of a far smaller proportion of TGCT case-series (4 of 7, Figure 1D).

### Overall CO-TGCT association and effects of study design

We estimated sRR of developing TGCT following a history of CO to be 4.0(95% *CI* = 3.4–4.6) in case-control studies (**Table S2Ai**) and 4.8(95% *CI* = 3.2–7.2) in cohort studies (**Table S2Bi**). The sRR estimated by pooling these results was 4.1(95% *CI* = 3.6–4.7), with 14% of the variance (I^2^) attributed to between-study heterogeneity (Figures 1A,B; Table **S2Ci**). By contrast, sRR estimated from studies that compared CO frequencies between TGCT case series and external populations was 14.8(95% *CI* = 10.7–20.4), with 86% of variance attributed to between-study heterogeneity (Figures 1C,D; **Table S2Di**).

Two additional features of study design appeared to modify sRR estimated among case-control and cohort studies: time CO was recorded, and inclusion of spontaneous descent in CO definition. The RR estimate was notably greater, 9.9(95% *CI* = 3.5–28.2), in the single study (Mills et al., 1984) that determined CO history from medical record entries made around the time of TGCT diagnosis, and substantially lower, 1.3(95% *CI* = 0.7–2.5), in the single study (Sabroe and Olsen, 1998) in which CO had been recorded at birth. This latter study was likely the only one to have included in the CO group a large proportion of men whose testes would have descended in the first months of life. The same study appeared to be largely responsible for modification by whether definition of CO included spontaneous descent (*p*_heterogeneity_ = 0.002 for analysis including this study, *p*_heterogeneity_ = 0.57 for analysis excluding it, **Table S2Cvi**). Accordingly, this study was a clear outlier in the funnel plot (Figure 1B), while all others fell very near or within the 95% confidence limits.

### Modification by features of cryptorchidism

Several features of CO appeared to modify the CO-TGCT association (Figure 2; **Table S3**). Analyses stratified on laterality of undescended testes relative to TGCT revealed an apparent trend: sRR estimates increased steadily in progression from TGCT arising in testes contralateral to unilateral CO [sRR = 1.5(95% *CI* = 0.9–2.6)], on unspecified side relative to unilateral CO [sRR = 3.5(95% *CI* = 2.7–4.7)], ipsilateral to unilateral CO [sRR = 5.4(95% *CI* = 3.7–7.8)], and in either testis following bilateral CO [sRR = 9.8(95% *CI* = 6.2–15.7)] (Figure 2A; **Table S3Ci**). Data on anatomic location of undescended testes, provided for no case-control study and only one cohort study (Swerdlow et al., 1997), were insufficient to determine whether TGCT risk differs appreciably following abdominal vs. inguinal non-descent (**Table S3Cii**). Therefore, the meta-analysis did not confirm the report from a single TGCT case series (Stone et al., 1991) of far higher RR following abdominal non-descent (**Table S3Dii**).

In analyses stratified on means of resolving CO, estimates increased in the progression of testes that descended spontaneously [sRR = 1.3(95% *CI* = 0.9–1.8)], testes repositioned by surgery or hormone therapy [sRR = 4.7(95% *CI* = 3.7–6.1)], and testes remaining undescended [sRR = 6.2(95% *CI* = 3.7–10.4)] (Figure 2B; **Table S3Ciii**). Analyses stratified on both means of resolution and age at resolution suggested greater RR among those with older age at spontaneous descent or therapeutic resolution, although estimates for those who experienced spontaneous descent did not achieve statistical significance (Figure 2C and **Table S3Civ**).

### Modification by features of TGCT

Analyses relating CO to tumor histology were conducted first in a subset of data excluding three studies that reported “no difference” in RR of seminoma vs. non-seminoma without providing stratum-specific estimates. The results hinted that RR of seminoma may exceed that of non-seminoma (**Table S4Ci**). To minimize the possibility that systematic omission of data from studies reporting no difference had spuriously created this impression, we repeated this analysis using a fuller set of data that included values imputed for those studies; these results also suggested a stronger association of CO with seminoma (Figure 3A; **Table S4Cii**).

Analyses within strata defined by year of TGCT diagnosis suggest that magnitude of the CO-TGCT association diminished steadily from the 1950's forward. Highest sRR, 6.8(95% *CI* = 4.2–10.9), was estimated among studies with midpoint year of TGCT diagnosis in the 1950's, and lowest, 3.6(95% *CI* = 3.1–4.1), in studies with midpoint 1990 or later (*p*_trend_< 0.001; Figure 3B; **Table S4Ciii**). Further stratification on histology, possible only for 1960's forward, revealed that this decrease results largely from a dramatic decrease in the CO-seminoma association [sRR = 9.3(95% *CI* = 4.0–21.8) for 1960–1969, sRR = 3.4(95% *CI* = 2.5–4.5) for 1990+; *p*_trend_ = 0.009), in contrast to no clear trend for other histologies (*p*_trend_= 0.320) (Figure 3C; **Table S4Civ**).

### Trends in occurrence of individual conditions

Analyses of frequency of CO among controls participating in the case-control studies, (Morrison, 1976; Henderson et al., 1979; Loughlin et al., 1980; Schottenfeld et al., 1980; Coldman et al., 1982; Depue et al., 1983; Mills et al., 1984; Pottern et al., 1985; Moss et al., 1986; Swerdlow et al., 1987; Gershman and Stolley, 1988; Strader et al., 1988; Haughey et al., 1989; United Kingdom Testicular Cancer Study Group (UK), 1994; Gallagher et al., 1995; Davies et al., 1996; Moller et al., 1996; Prener et al., 1996; Petridou et al., 1997; Sabroe and Olsen, 1998; Weir et al., 2000; Stang et al., 2001; Bonner et al., 2002; Herrinton et al., 2003; Dieckmann and Pichlmeier, 2004; Hardell et al., 2007; McGlynn et al., 2007; Walschaerts et al., 2007; Dusek et al., 2008; Lacson et al., 2012) for which midpoint TGCT diagnosis years ranged from 1960's through 1990+, revealed no evidence that CO became more common among TGCT-free men during this period (*p*_trend_ = 0.295, data not shown).

Separate analyses of SEER 9 incidence data revealed that while annual age-standardized incidence of non-seminoma/mixed GCT increased over 28% (from 1.8 to 2.3 new diagnoses per 100,000) from 1973–1978 to 1994–1998, the increase in seminoma was far greater, 68% (from 2.2 to 3.7 per 100,000) during the same interval (**Figure S2**).

## Discussion

Meta-analysis of data from case-control and cohort studies suggests that young men with a history of CO experience approximately 4-fold increased risk of TGCT. Estimates from TGCT case-series whose frequency of CO was compared with separate populations were regarded as unreliable; this practice may cause severe bias (Rothman et al., 2008), which may account for the far larger sRR and between-study variation estimated from these reports.

Addressing more subtle differences in study design, we found that sRR estimates from case-control and cohort studies were notably modified by methods used to determine a man's history of CO. The stronger association estimated in the single case-control study in which history of CO was determined from medical record notes made at TGCT diagnosis may have arisen from better recall of CO among cases than controls, since CO was a recognized TGCT risk factor when these diagnoses were made. Such recall bias may have contributed, also, to large effects reported for case series, because history of CO was similarly determined in all of these studies. Undescended testes that descended spontaneously in infancy would rarely be recorded in medical records after the neonatal period, or recalled by study participants or their mothers. We therefore anticipate that only the single study in which CO was recorded at birth (Sabroe and Olsen, 1998) would have identified a high proportion of men with this history. Weak CO-TGCT association reported in this study may indicate that boys born with CO whose testes descend spontaneously in early months experience TGCT risk approaching that of the general population. This possibility has implications for both managing CO and understanding origins of TGCT, so we hope that it will be explored further in retrospective cohorts for which there are detailed neonatal records.

The apparent modification of sRR by features of CO and TGCT may provide insight regarding TGCT etiology. Two general explanations for the CO-TGCT association have long been offered. The *common cause hypothesis* attributes the association to one or more unidentified etiologic factors shared by CO and TGCT, whereas the *position hypothesis* asserts that suprascrotal environment increases malignant potential of undescended testes. Hussman suggested two testable predictions of the position hypothesis (Husmann, 2005): (1) in unilateral CO, fully descended contralateral testes should not experience elevated TGCT risk, and (2) early orchiopexy should decrease TGCT risk. Regarding the first, we estimated a lesser sRR for contralateral testes [1.5(95% *CI* = 0.9–2.6)] than for ipsilateral testes [5.4(95% *CI* = 3.7–7.8)], suggesting a deleterious effect of suprascrotal position. Also consistent with positional effects, sRR among those with bilateral non-descent [9.8(95% *CI* =6.2–15.7)] was, within statistical precision of the meta-analysis, indistinguishable from twice sRR of those with unilateral non-descent [2×sRR_ipsilateral_ = 10.8(7.4–15.6)]. However, published data are insufficient (24 cases in 7 studies) to rule out a small increase in risk to contralateral testes (e.g., 20%), as would be required to strictly affirm Hussman's first prediction. We note, however, that some true increase in risk to contralateral testes would not necessarily rule out the position hypothesis, because central responses to a single testis in a suprascotal position could, in theory, contribute to malignant potential of the contralateral testis. For example, in rodent models of unilateral CO created surgically, degenerative changes (Quinn, 1991; Zakaria et al., 1998) and altered gene expression (Iizuka et al., 1996) were demonstrated in contralateral, descended testes. A phenomenon observed in humans is also consistent with this possibility: among patients with unilateral TGCT who undergo biopsy of the contralateral testis, men with a history of CO are more often found to have the presumptive TGCT precursor carcinoma *in situ* testis/intra-tubular germ cell neoplasia than those without history of CO (Dieckmann and Loy, 1996). Regarding Hussman's second prediction, we observed greater TGCT risk among those who experienced later resolution. However, we cannot confidently conclude that deleterious effects of suprascrotal position are responsible. An alternate explanation, which we cannot rule out, is that men with earlier resolution of CO experienced as a group inherently lower risk of TGCT. This might occur, for example, if this group included a higher proportion of boys destined to experience spontaneous descent if therapeutic intervention had been delayed. Because elevated risk was observed, regardless of age at orchiopexy, the clinical significance of available data is that patients undergoing orchiopexy at any age should be closely monitored; thus along with their parents or primary care givers and primary care physicians, they should be made aware of the increased risk.

Unfortunately, published data could not distinguish between risks experienced by men with histories of abdominal vs. inguinal non-descent. These distinct phenotypes may provide a means of determining relevance to human CO and TGCT of animal models of CO with high (Hsieh-Li et al., 1995; Rijli et al., 1995; Good et al., 1997; Nef and Parada, 1999; Overbeek et al., 2001) or low (Hutson, 1986; Lahoud et al., 2001) non-descent resulting from disruption of specific genes. If found to be relevant to human CO and TGCT, these models may become valuable tools in TGCT research, which has long suffered from absence of animal models of common forms of TGCT (Oosterhuis and Looijenga, 2005). Therefore, documenting position of undescended testes before orchiopexy may be useful for future research and in follow-up of men with a history of CO, particularly in the era of electronic medical records.

Modification by features of TGCT may suggest etiologic heterogeneity of these tumors.

Morrison (1976) apparently first suggested that CO is more strongly associated with seminoma than non-seminoma, although subsequent reports were inconsistent. The meta-analysis suggested greater RR for seminoma than for non-seminoma, and an intermediate value for mixed GCT, by addressing a far larger set of data than any single study and treating mixed GCT as a distinct histologic type. Although mechanisms underlying this pattern remain unknown, this finding suggests that tumors of distinct histologic types may have separate etiologies and/or result from events at different developmental stages. This possibility accords with the far more dramatic decreases in the CO-seminoma association observed over time. This trend, together with the observation that incidence of seminoma has risen more rapidly than that of non-seminoma/mixed GCT (**Figure S2**), raises the intriguing possibility that increasing occurrence of seminoma may involve increasing exposure to unidentified environmental factors through processes unrelated to CO.

In conclusion, this meta-analysis provides a detailed quantitative summary of available high-quality observational data on the association between CO and TGCT, including observations that can no longer be made due to trends toward younger age at CO repair and increasing TGCT incidence. Results of subgroup analyses indicate possible future directions in understanding both stratification of TGCT risk among boys born with undescended testes and TGCT etiology. Meta analyses addressing features of CO suggest that while bilateral CO is associated with nearly twice the TGCT risk as unilateral CO, data are inadequate to assess the role of anatomic position of the undescended testis. Early repair is associated with lower TGCT risk, but published data do not provide a basis for recommending optimal time of repair or to determine whether optimal repair can reduce risk to baseline. Therefore, all CO patients and their families should be counseled to be aware of future risk. Additional data are needed to affirm the possibility that TGCT risk is not elevated among boys whose testes descend spontaneously after birth. Results of subgroup analyses addressing features of TGCT suggest multiple pathways to malignancy and indicate considerable heterogeneity in risk of TGCT following CO. Both possibilities warrant mechanistic examination using contemporary tools of molecular biology.

## Dedication

The authors dedicate this report in memory of the life and work of our esteemed colleague, Dr. Brian E. Hardy.

### Conflict of interest statement

The authors declare that the research was conducted in the absence of any commercial or financial relationships that could be construed as a potential conflict of interest.
